# Natural experiments and long-term monitoring are critical to understand and predict marine host–microbe ecology and evolution

**DOI:** 10.1371/journal.pbio.3001322

**Published:** 2021-08-19

**Authors:** Matthieu Leray, Laetitia G. E. Wilkins, Amy Apprill, Holly M. Bik, Friederike Clever, Sean R. Connolly, Marina E. De León, J. Emmett Duffy, Leïla Ezzat, Sarah Gignoux-Wolfsohn, Edward Allen Herre, Jonathan Z. Kaye, David I. Kline, Jordan G. Kueneman, Melissa K. McCormick, W. Owen McMillan, Aaron O’Dea, Tiago J. Pereira, Jillian M. Petersen, Daniel F. Petticord, Mark E. Torchin, Rebecca Vega Thurber, Elin Videvall, William T. Wcislo, Benedict Yuen, Jonathan A. Eisen

**Affiliations:** 1 Smithsonian Tropical Research Institute, Balboa, Ancon, Republic of Panama; 2 UC Davis Genome Center, University of California, Davis, Davis, California, United States of America; 3 Marine Chemistry and Geochemistry Department, Woods Hole Oceanographic Institution, Woods Hole, Massachusetts, United States of America; 4 Department of Marine Sciences and Institute of Bioinformatics, University of Georgia, Athens, Georgia, United States of America; 5 Department of Natural Sciences, Manchester Metropolitan University, Manchester, United Kingdom; 6 Tennenbaum Marine Observatories Network, Smithsonian Environmental Research Center, Edgewater, Maryland, United States of America; 7 Department of Ecology, Evolution and Marine Biology, University of California Santa Barbara, Santa Barbara, California, United States of America; 8 Smithsonian Environmental Research Center, Edgewater, Maryland, United States of America; 9 Gordon and Betty Moore Foundation, Palo Alto, California, United States of America; 10 Department of Biological, Geological and Environmental Sciences, University of Bologna, Bologna, Italy; 11 Centre for Microbiology and Environmental Systems Science, University of Vienna, Vienna, Austria; 12 Department of Microbiology, Oregon State University, Corvallis, Oregon, United States of America; 13 Center for Conservation Genomics, Smithsonian Conservation Biology Institute, Washington, DC, United States of America; 14 Department of Ecology and Evolutionary Biology, Brown University, Providence, Rhode Island, United States of America; 15 Department of Evolution and Ecology, University of California, Davis, Davis, California, United States of America; 16 Department of Medical Microbiology and Immunology, University of California, Davis, Davis, California, United States of America

## Abstract

Marine multicellular organisms host a diverse collection of bacteria, archaea, microbial eukaryotes, and viruses that form their microbiome. Such host-associated microbes can significantly influence the host’s physiological capacities; however, the identity and functional role(s) of key members of the microbiome (“core microbiome”) in most marine hosts coexisting in natural settings remain obscure. Also unclear is how dynamic interactions between hosts and the immense standing pool of microbial genetic variation will affect marine ecosystems’ capacity to adjust to environmental changes. Here, we argue that significantly advancing our understanding of how host-associated microbes shape marine hosts’ plastic and adaptive responses to environmental change requires (i) recognizing that individual host–microbe systems do not exist in an ecological or evolutionary vacuum and (ii) expanding the field toward long-term, multidisciplinary research on entire communities of hosts and microbes. Natural experiments, such as time-calibrated geological events associated with well-characterized environmental gradients, provide unique ecological and evolutionary contexts to address this challenge. We focus here particularly on mutualistic interactions between hosts and microbes, but note that many of the same lessons and approaches would apply to other types of interactions.

## Abstract 2

This Essay argues that in order to truly understand how marine hosts benefit from the immense diversity of microbes, we need to expand towards long-term, multi-disciplinary research focussing on few areas of the world’s ocean that we refer to as “natural experiments,” where processes can be studied at scales that far exceed those captured in laboratory experiments.

## Main

It is widely recognized that host-associated microbes play profound roles in the health of their marine hosts and the ecosystems they inhabit. Although some such interactions with microbes are transient, many are more persistent and can be generally described as symbioses. Symbioses come in many flavors including parasitism, commensalism, and mutualism (see Box 1), and, in this paper, we focus in particular on the mutually beneficial (i.e., mutualistic) subset of such interactions involving marine hosts. Despite the wide recognition of the importance of such mutualisms, it remains less clear how these associations scale up to drive broader ecological and evolutionary patterns and processes. For example, the contribution of microbes to host acclimatization and adaptation (see Box 1 for definitions) is an active new field of experimental research with much potential. Studies, mostly conducted in controlled laboratory settings, have evaluated the ecological costs/benefits for hosts to associate temporarily with different microbes (e.g., corals [1–4]) or to engage in obligate intimate relationships (e.g., bobtail squid with the bioluminescent bacteria *Aliivibrio fischeri* [5]).

**Box 1. Definitions of key terms**

Acclimatization: The process by which an organism becomes accustomed to new environmental conditions during its lifetime.

Adaptation: A heritable trait of an organism that increases its fitness in its surrounding environment. In comparison to acclimatization, adaptations will be passed on to the next generation.

Convergent evolution: Independent origins of similar features in different organisms in response to separately experiencing similar selective pressures. Importantly, convergently originated features, also known as analogous features, were not present in the common ancestor of the taxa in question.

Genetic drift: Change in the relative frequency of genotypes due to random variation in reproduction. Such drift is more common in small populations and leads to changes in genotype frequencies independent of adaptive forces.

Host–microbe coevolution: During host–microbe coevolution, multicellular hosts and their associated microbes show a concerted and heritable response to an environmental change.

Homologous recombination: The process by which two pieces or stretches of DNA that are very similar in their sequence physically align and exchange nucleotides.

Horizontal gene transfer: The unidirectional movement of DNA, usually only small fractions of a genome, from one organism to another. Though this generally occurs more frequently within species than between, it can also occur across vast evolutionary distances.

Metagenomics: Studies of the genetic material of communities of organisms.

Phenotypic plasticity: Phenotypic plasticity is the ability of a specific genotype to produce more than one phenotype in response to a changing environment during an individual’s lifetime. These phenotypic changes may include an organism’s behavior, morphology, physiology, or other features. Phenotypic plasticity is adaptive if it increases an individual’s survival and if the ability is passed on to the next generation.

Symbioses: Symbioses are broadly defined as intimate interactions between at least two organisms where at least one of them benefits. We focus here specifically on mutually beneficial interactions (aka mutualisms) between multicellular eukaryotes and their associated microbes. These interactions may include disease resistance, predator avoidance, and nutrition. These interactions will ultimately increase host survival and fitness.

Experimental studies are, however, intrinsically limited in several ways. They limit themselves to a small number of experimentally tractable hosts and microbes, and, in doing so, fail to account for the enormous complexity of interactions and variation that exist in nature between multiple hosts and their multitudes of associated microbes. Short-lived experiments (e.g., days to weeks) cannot replicate the scales of time and space involved in the potential coevolution of hosts and microbes (Box 1). Attempts to merge long-term datasets to reveal overarching patterns (e.g., [6–9]) have provided valuable insights but are shadowed by the limits and biases introduced by mixing information from different contexts or methodologies [10]. These limitations obscure general principles on the roles (mutualistic or otherwise) of host-associated microbes across host individuals, species, and communities [11–13].

**Fig 1:**
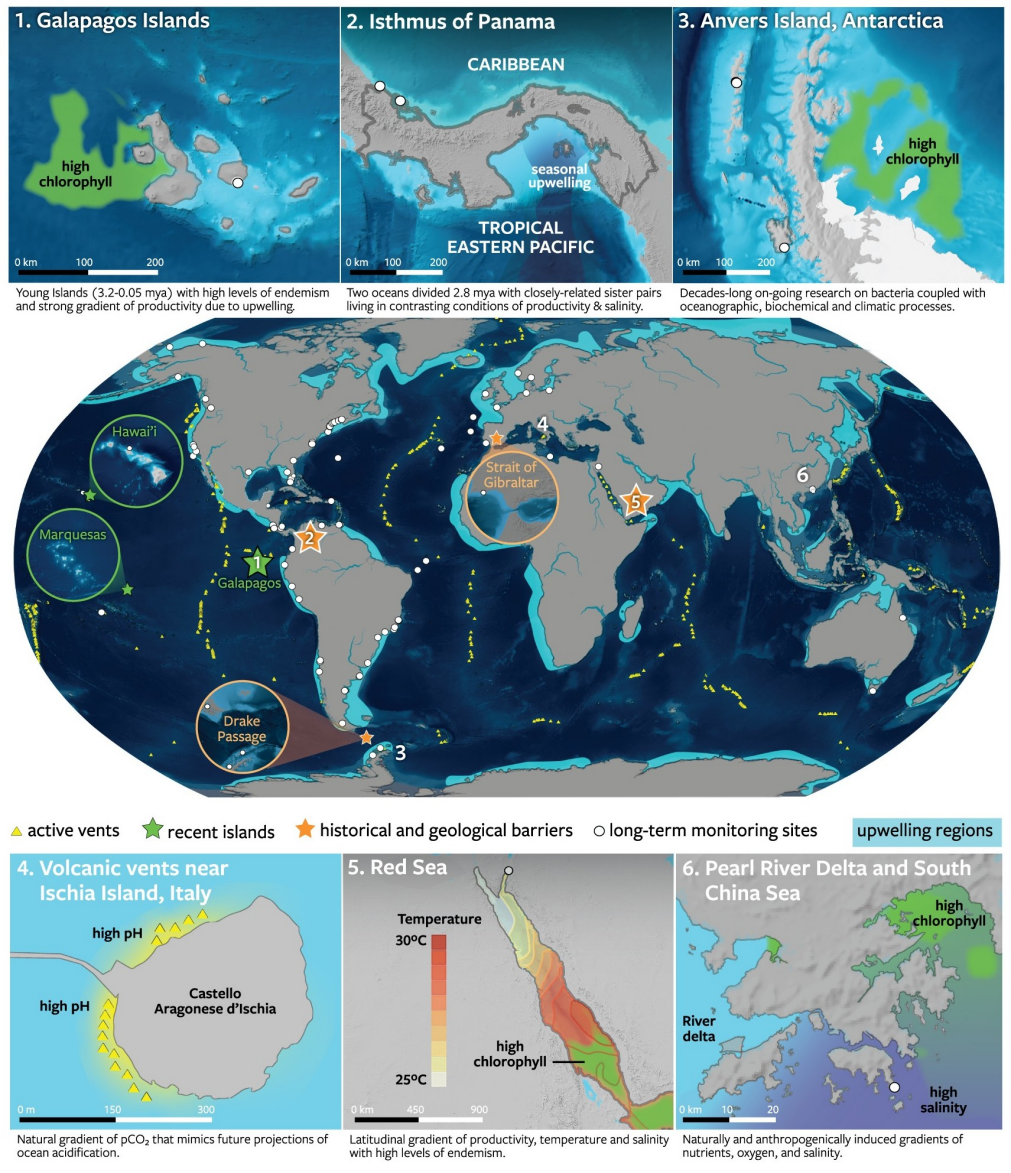
Examples of marine natural experiments as observatories of host–microbe interactions. Regionally focused, long-term, and taxonomically broad research programs will help fill key knowledge gaps about the nature of microbe functions and the dynamics of host–microbe interactions in changing oceans. We highlight areas of the world’s oceans where environmental gradients are well characterized, where the taxonomy and evolutionary history of the local host fauna and flora is already well established, where paleoecological studies can provide important historical context, where a long-term monitoring program is ongoing, and where there is significant research infrastructure. Long-term monitoring sites (white dots) include sites of the NSF’s LTER Network, the Smithsonian Institution’s MarineGEO network of partners, the MBON, the AIMS, and the ASSEMBLE. (1) NASA MODIS data; (2) Adapted from [93]; (3) Adapted from [73]; (4) Adapted from [74]; (5) Adapted from [94]; (6) Adapted from [95]. AIMS, Australian Institute of Marine Science; ASSEMBLE, Association of European Marine Biological Laboratories; LTER, Long-Term Ecological Research; MarineGEO, Marine Global Earth Observatory; MBON, Marine Biodiversity Observation Network.

Here, we demonstrate the value of moving beyond taxon-centric approaches to studying host–microbe associations in their natural evolutionary and ecological context. We suggest intensifying long-term research in well-documented “natural experiments”. Such natural experiments, including well-calibrated geological events (e.g., vicariance and creation of novel habitats accurately dated using fossil and geological data) and environmental gradients where multiple hosts and associated microbes are subjected to the same range of environmental conditions, can be particularly useful (Fig 1). These phenomena provide a unique framework for comparative studies where the processes of interest occur over spatial and evolutionary time scales that are nearly impossible to capture in laboratory experiments. The value of combining experimental and long-term field studies at natural experiments has been recognized by ecologists [14–16]. We argue that similar approaches should be applied to the study of host–microbe interactions. We highlight several natural experiments that can advance our understanding of the ecological and evolutionary mechanisms shaping host–microbe interactions (with a focus on mutualistic ones) in marine communities and ecosystems.

### Identifying important players

Marine organisms have evolved complex structural, behavioral, and chemical mechanisms to regulate the presence, abundance, and activity of their microbial associates. Hosts can limit colonization by transient opportunistic microbes that would use space and resources without providing any benefits, and some hosts can even block pathogens entirely [17–19]. Host-specific and obligate microbial associates, often called the “core microbiome” of a host population or species, are generally assumed to play more important functional roles than opportunistic and transient taxa [20]. This core microbiome is exemplified by an obligate nutritional microbial symbiosis, in which the host relies extensively on microbial partners for survival by synthesis of food, often in a nutrient-limited habitat. The host may acquire these partners horizontally (from the surrounding environment), vertically (from the parent to the offspring), or in both ways (mixed mode) [21]. Many evolved symbioses result in codependency; for example, the genomes of host-associated microbes have lost genes encoding pathways that were previously essential, such as those for motility or environmental stress responses, but that became obsolete in obligate symbiotic lifestyles [22]. In return, hosts have evolved mechanisms to maintain their associated microbes in stable intracellular environments and to support their nutritional needs [23]. Some of these nutritional associations are clearly identifiable because symbionts form massive and dense populations, sometimes only consisting of a single microbial species, in or on the bodies of their hosts. Examples include photosynthetic symbioses in cnidarians [24] and chemosynthetic symbioses in invertebrate animals such as bathymodiolin mussels, lucinid clams, *Riftia* tubeworms, and *Astomonema* nematodes [25,26]. Although widespread, host reliance on a single or few microbes for nutrition are the exception rather than the rule. The vast majority of animals and plants are instead associated with a diverse assemblage of microbes where it is challenging to differentiate between members of the core microbiome and the myriad of transient microbes and even more challenging to determine what, if any, key functional roles such microbes play.

Several approaches have been proposed to identify key microbes or functions within complex host microbiomes (reviewed in [27]). The most common practice is to identify microbial taxa that are consistently associated with a host population or species using marker gene sequencing, usually above some arbitrary prevalence threshold ([28]; but see [29,30] for alternative methods). The prevalence of a host–microbe association is typically measured without explicit attention to co-occurring and closely related host taxa, the surrounding environment, or adequacy of spatial and temporal sampling. This limited sampling and lack of context, often resulting from funding constraints, leads to several major limitations. First, a microbial taxon can be prevalent in a host population for reasons unrelated to its functional role. For example, it may originate from the host’s food or habitat, including seawater or sediment [31]. Second, even the core microbiome can change over time [32]. Functionally important microbes may fluctuate in abundance throughout host ontogeny and may also vary seasonally. Essential host-associated microbes may be overlooked if the sampling method cannot detect low abundance reliably, resulting in false negatives, or if sampling is sporadic, missing the life stage or season when particular microbes are essential. Third, many studies rely upon sequencing of rRNA genes to characterize communities, yet rRNA genes are generally too conserved to distinguish closely related taxa and reveal little directly about genomic functional potential. Clearly, understanding the functional roles of host-associated microbes requires analyses that go far beyond individual marker gene profiles and instead encompass other types of information such as whole genomes or metagenomes, transcriptomes, metabolomes, localization, biochemistry, and more. Fourth, taxon-focused studies may miss valuable information about interactions that could be gleaned from broader comparative analyses. Microbes that are specific to particular host genotypes, host species, or closely related groups of hosts, indicating a shared evolutionary history, are likely candidates for core microbes with specialized functions (e.g., gut fermenters associated with herbivores). These existing limitations could be robustly circumvented via whole-ecosystem studies where long-term collection of comprehensive genomic-level datasets (e.g., ‘omic scale information) would transform our understanding of host–microbe interactions at all levels.

To instigate this new approach, we recommend strategically intensifying research within a few ocean regions. This entails collecting large scale data on host-associated microbes across phylogenetically diverse sets of co-occurring host organisms, together with data on surrounding free-living microbes (i.e., in seawater and sediments) through time in areas where the surrounding abiotic environment and community dynamics have been well characterized. A regionally focused and coordinated approach will allow identifying environmental sources and hosts that serve as reservoirs of key host-associated microbial taxa and genes. Long-term investments in research on particular communities of hosts and microbes will also help establish links between changes in core microbiome composition, environmental factors, ecosystem function, and resilience. Public archival of genomic data and samples (available for complementary analysis using emerging technologies) collected from a few intensively studied ocean regions will foster transformative discoveries on dynamic host–microbe relationships. Habitat-forming corals, sponges, seagrasses, and mangrove trees are important focal groups, since breakdowns in the associations between these species and their microbiomes likely disproportionately influence other taxa and ecosystem functions. However, this should not come at the expense of research on more inconspicuous and overlooked, yet functionally important taxa that comprise the majority of the oceans’ biological diversity (e.g., small fish that fuel marine food webs [33] and urchins and crustaceans that feed on algae that can displace corals [34]). Systematic biases toward studying certain taxa (vertebrates, species with large body sizes, charismatic fauna), partly caused by the lack of coordination, have clearly affected our understanding of the distribution and roles of host-associated microbes. For example, a recent microbiome comparison of several Indo-Pacific invertebrate species demonstrated that sponges have a less specific microbiome than had been assumed for many years [35]. Expanding the taxonomic breadth of host–microbe studies will be most fruitful in areas where taxonomically rigorous field guides, ecological survey data, and functional trait databases are available. Substantial progress will also occur where phylogenetic relationships are known and local expert taxonomists can be engaged. One of the numerous potential outcomes includes building community-wide association matrices to unveil the extent of reliance between hosts and microbial partners (specificity versus ubiquity, obligate versus facultative) and the interactions that promote the stability of core microbiomes.

### Role of microbes in host acclimatization and adaptation

**Fig 2:**
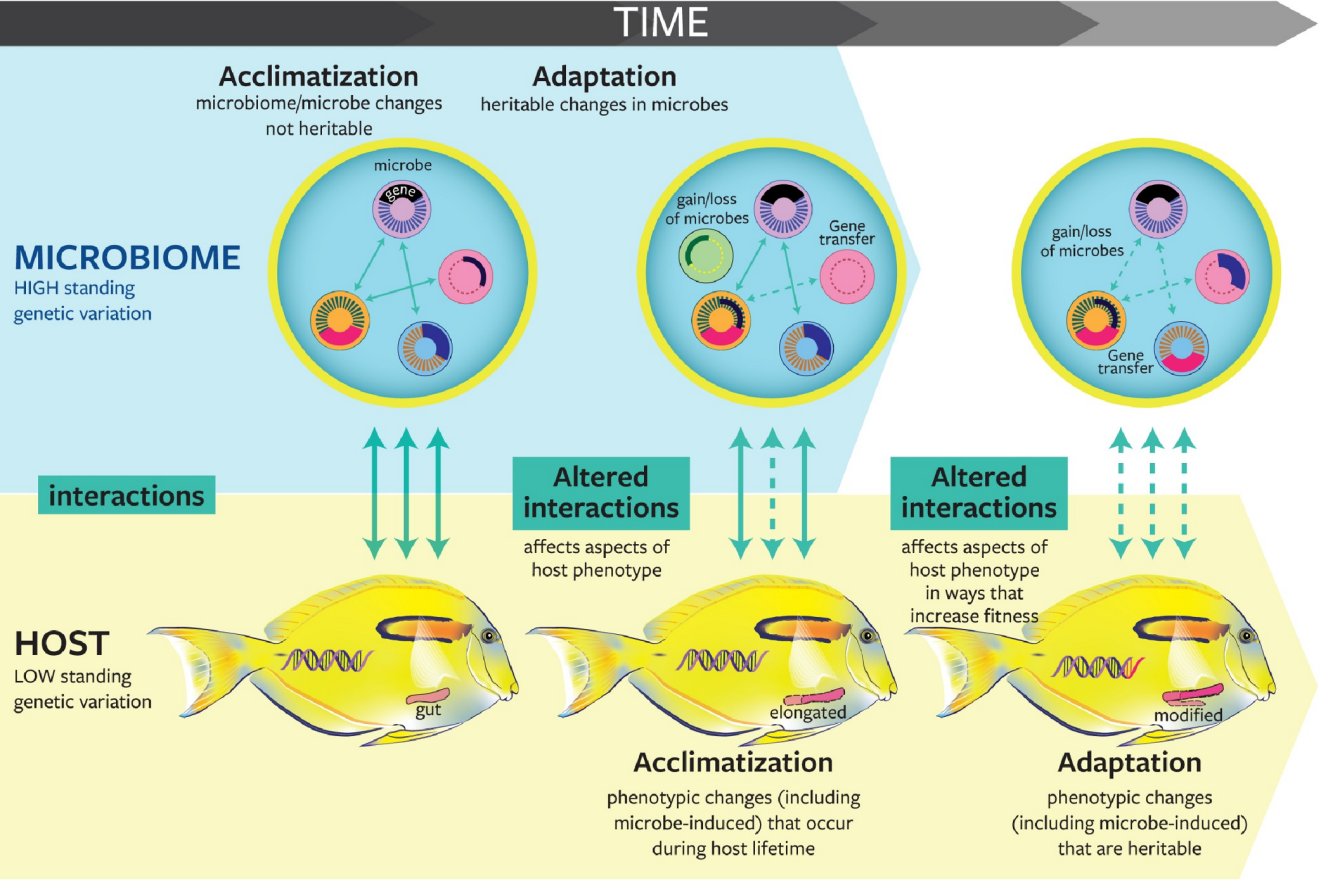
Conceptual representation of the role of microbes in host acclimatization and adaptation. Microbes can frequently adapt to environmental changes more rapidly than their host because of shorter generation times and higher standing genetic variation. Changes that occur at the levels of individual microbes and microbiomes can rapidly generate phenotypic plasticity in a broad range of host traits (i.e., one host genotype expresses multiple phenotypes induced by microbes). Microbially induced phenotypes may promote host adaptation if they become heritable traits. Within microbiomes, transient microbes (thin dashed circles) have limited effects on host phenotype. On the other hand, core microbes (thick dashed circles) that engage in prolonged relationships with hosts and potentially coevolve with hosts likely alter host phenotypes and promote host adaptation. Note that the time scale at which evolutionary changes occur varies widely between organisms, but adaptation is generally slower than acclimatization. Plain line: nonaltered interaction; dashed line: altered interaction; colors of microbes represent different microbial taxa.

Host-associated microbes can rapidly respond to extrinsic factors such as extreme or anomalous environmental conditions (e.g., heatwaves, hypoxia), pathogens, anthropogenic disturbances (e.g., pollution, overfishing, aquaculture, invasive species), and acute and chronic stressors [36,37]. They can also quickly change in response to factors intrinsic to the host (e.g., changes in host physiology [38]). The dynamic nature of microbes may provide a source of ecological and evolutionary novelty to support potential host response mechanisms that augment the host’s own evolutionary potential. Host-associated microbial communities can shift rapidly through the loss, gain, or replacement of individual members. Individual microbial cells can make rapid physiological adjustments during their lifetime (plasticity) or within a few generations (adaptation) [39] (Fig 2). In many microbes, relatively high rates of mutation and exchange of genetic material among divergent lineages (through homologous recombination and horizontal gene transfer) generate a high frequency of new genetic variants, some of which may be better suited to novel conditions (Fig 2). These mechanisms contribute to fueling an immense standing pool of genetic variation that hosts can potentially draw upon. The outcomes of the collective ecological and evolutionary response of hosts and their associated microbes to environmental change may comprise 1 of 4 nonmutually exclusive scenarios [40,41]: (1) Imbalance: a temporary or permanent change of host fitness and microbial functions leading to increased disease susceptibility; (2) Resistance: the microbiome continues performing its functions and the host does not lose or gain fitness; (3) Acclimatization: the newly formed microbial community in conjunction with host phenotypic plasticity enable the individual host to adjust and maintain performance under changing environmental conditions (Fig 2); and (4) Adaptation: in the long term, newly formed interactions between host genotypes and associated microbes increase the fitness of the symbiosis and they become heritable (Fig 2).

**Fig 3:**
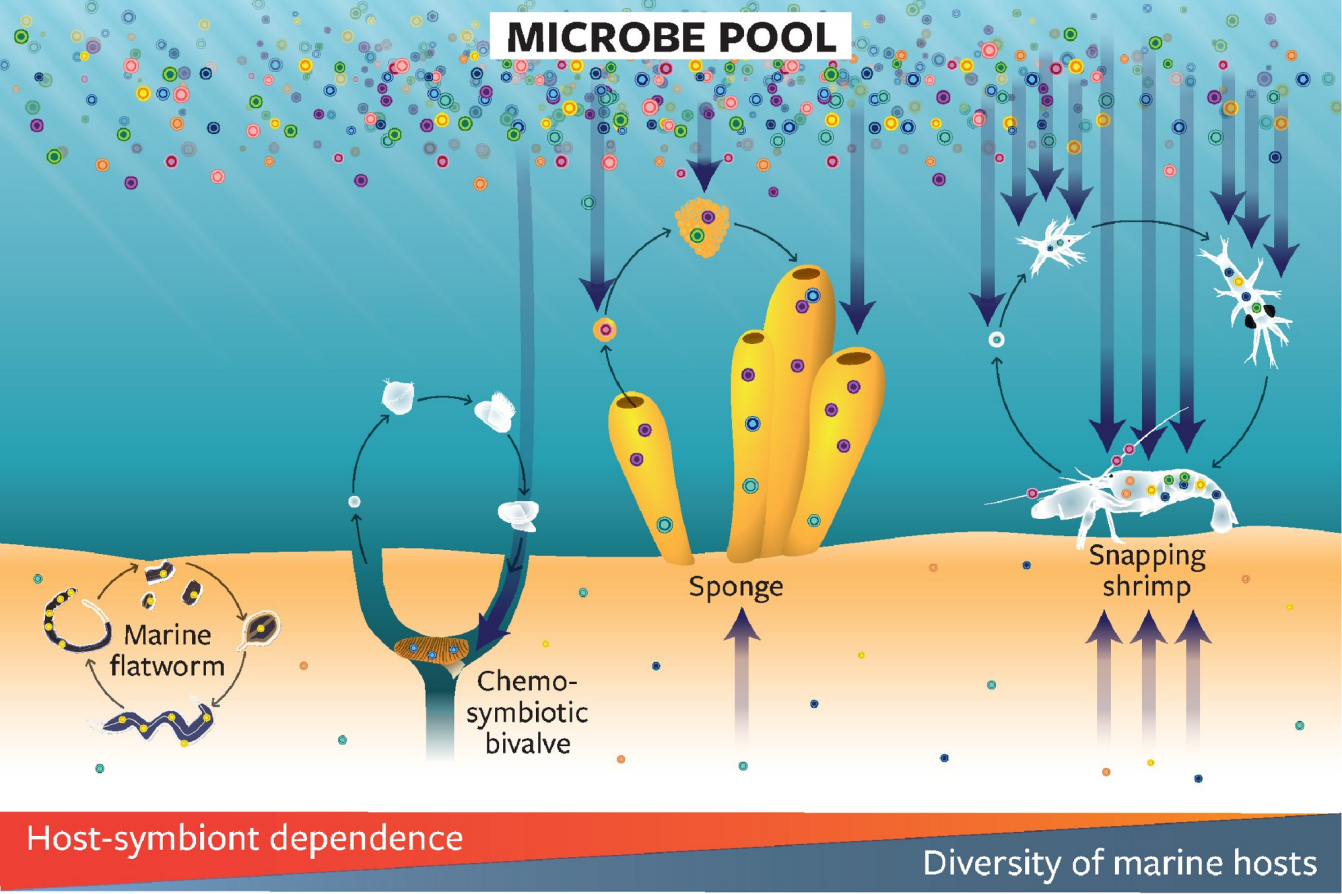
The role of microbes in the host’s response to environmental changes is contingent upon their predominant mode of transmission. Microbes that are present in the marine environment represent a vast pool of standing genetic variation. The majority of marine species with horizontal (e.g., lucinid clams and snapping shrimps) or mixed mode of symbiont acquisition (e.g., sponges) interact with a large number of microbes that they acquire during their lifetime. The ability to draw on this large evolutionary potential by switching microbes or gaining new genes potentially allows hosts to respond rapidly to environmental changes. At the other end of the spectrum, the few marine hosts with strictly vertically transmitted symbionts (e.g., flatworms) have less opportunity to exchange genes to rapidly adjust the symbiosis to changing conditions.

The role that host-associated microbes play in their host’s response to environmental change is also influenced by their mode of transmission (Fig 3). While vertical transmission may help ensure the intergenerational stability of mutualistic symbioses, the dependence on symbionts with highly simplified and inflexible genomes is a risky strategy under variable or unpredictable stressful conditions [42,43]. Vertically transmitted symbionts have fewer opportunities to exchange genes with the vast pool of genetic diversity available in the external environment, which could constrain the adjustment of these associations to rapidly changing conditions. In the marine environment, the vast majority of mutualistic symbionts are acquired horizontally from the surrounding environment or from other hosts [44]; this includes associations where a host is entirely dependent upon a single or a few symbionts for nutrition (e.g., tubeworms [45]; mussels [46]). Horizontal transmission has important implications for the adaptive potential of hosts [47]. The ability to acquire microbes and genes from the surrounding environment allows hosts to access the huge evolutionary potential contained within the larger microbial communities. Hosts with horizontally acquired microbes could thus be better positioned to adjust and become resilient to changing environmental conditions. Selection that maintains and fine-tunes the relationship could subsequently lead to adaptive genetic change.

Several key bottlenecks currently impede our understanding of how host-associated microbes drive the initial response as well as long-term, evolutionary adaptation to climate change–related disturbances in hosts with diverse microbial communities. First, changes in microbiomes that confer adverse or beneficial outcomes for the host cannot be distinguished from natural variability without adequate measures of host phenotypes that covary with fitness. Unlike photosymbiotic organisms that exhibit quantifiable phenotypic responses to stress (e.g., using a bleaching index or symbiont density), the early signs of physiological stress are difficult to observe and measure in the vast majority of marine host–microbe associations. Second, studies are rarely designed to disentangle causes from effects. Before–after studies correlate seemingly altered microbial communities with perturbations or diseases, often without establishing causality in the relationship [48,49]. Third, most research in this field has been conducted over temporal scales that are not suited for understanding processes of acclimatization and adaptation that may occur over months to decades [50]. Single or multistressor laboratory experiments conducted over days to weeks are powerful means to identify environmental thresholds beyond which the host–microbiome interactions become disrupted [51]. However, how experimental results can be extrapolated to understand the response of natural systems exposed to ambient microbes and heterogeneous stressors in their natural environment remains unclear. Fourth, the response of host–microbe mutualistic symbioses to stressors is partly shaped by the environmental conditions experienced during the lifetime of the host and by previous generations, although that information is rarely considered or available. For example, the susceptibility of corals to future environmental changes is partly contingent upon changes in algal symbiont composition that occurred as a result of previous exposures to temperature anomalies (i.e., symbiont shuffling in the controversial adaptive bleaching hypothesis [52]). Therefore, the tolerance of hosts and their host-associated microbes to environmental change is rarely interpretable without ecological context [53]. Finally, there is a dearth of paired host and microbial genomes in public databases. The lack of population-wide data relating traits of interest to host and microbial genomic variation at the individual level (i.e., genome-wide association studies) limits our understanding of how genomic innovations contribute to host acclimatization and adaptation [54].

Bolstering our understanding of the mechanisms of host–microbe evolution requires investing resources into long-term multidisciplinary research on diverse communities of hosts and microbes distributed across well-characterized environmental gradients. Rigorously designed comparative population genomic studies and field experiments (e.g., reciprocal transplants) combined with measures of host phenotypes using methods such as in situ imaging [55], immunological assays [56], gene expression [57], metabolomic profiling [58], and behavioral assays [59] will illuminate adaptive genetic variants, how they are transferred among microbial strains across host communities, and their impacts upon host fitness. Repeated through time, these measures will provide unique insights into how microbiome-mediated phenotypic plasticity may allow hosts to rapidly accommodate to novel environments or resources (e.g., microbes allow some host individuals to obtain nutrients from novel foods) through periodic (e.g., seasonal fluctuations) and transient environmental changes (e.g., heat waves). For foundational, long-lived, and large colonial host species, noninvasive methods exist for repetitive sampling of tagged individuals (e.g., for corals [60]). The focus should also expand beyond foundation species to include small, ecologically important host organisms and those with life history strategies that make them particularly tractable for transgenerational studies. This approach will only be fruitful if integrated measures of hosts and microbiomes are collected over multiple generations (i.e., beyond the time scale of a typical scientific project), where physiochemical parameters are being monitored, and where the evolutionary history of the local host fauna and flora is already well established. Targeted comparative research can similarly leverage natural experiments that have played out over longer time scales. Sudden discontinuities in the distribution of many closely related populations and species have been linked to geological vicariant effects, sharp environmental gradients, or a combination of both [61]. Organisms on opposing sides of dispersal barriers (sometimes impassable) follow different evolutionary trajectories under the influence of local environmental conditions [62]. These systems provide unique historical contexts in which researchers can generate testable hypotheses about the role that host-associated microbes played in the evolution of host traits. Signatures of convergent evolution, evident at the ecosystem-wide level (i.e., similar patterns observed across many hosts and symbionts that have been exposed to similar selective pressures), likely reflect fundamental principles of adaptation [63].

### Examples of natural experiments

Natural experiments are past events or gradients that allow researchers to explore biological patterns and processes on spatial and temporal scales that far exceed those possible in the laboratory. Natural experiments may or may not be created or altered by humans and have been the bread and butter of natural historians, biogeographers, and evolutionary biologists for decades. Building on this substantial body of conceptual work, we propose that natural experiments can also enlighten our understanding of the evolution and ecology of host-associated microbes and their hosts. We present examples of natural experiments where the outcomes of complex interactions can be observed with replication to provide insights into the processes underlying host–microbe evolution. Our examples focus on well-characterized systems where host evolution has already been well explored, thereby allowing “tests” that approach the rigor of laboratory experiments. We expect that studying natural experiments like these will allow general principles of host–microbe evolution to emerge when repeated patterns are observed within a system or across different systems.

### Biogeography

**Fig 4:**
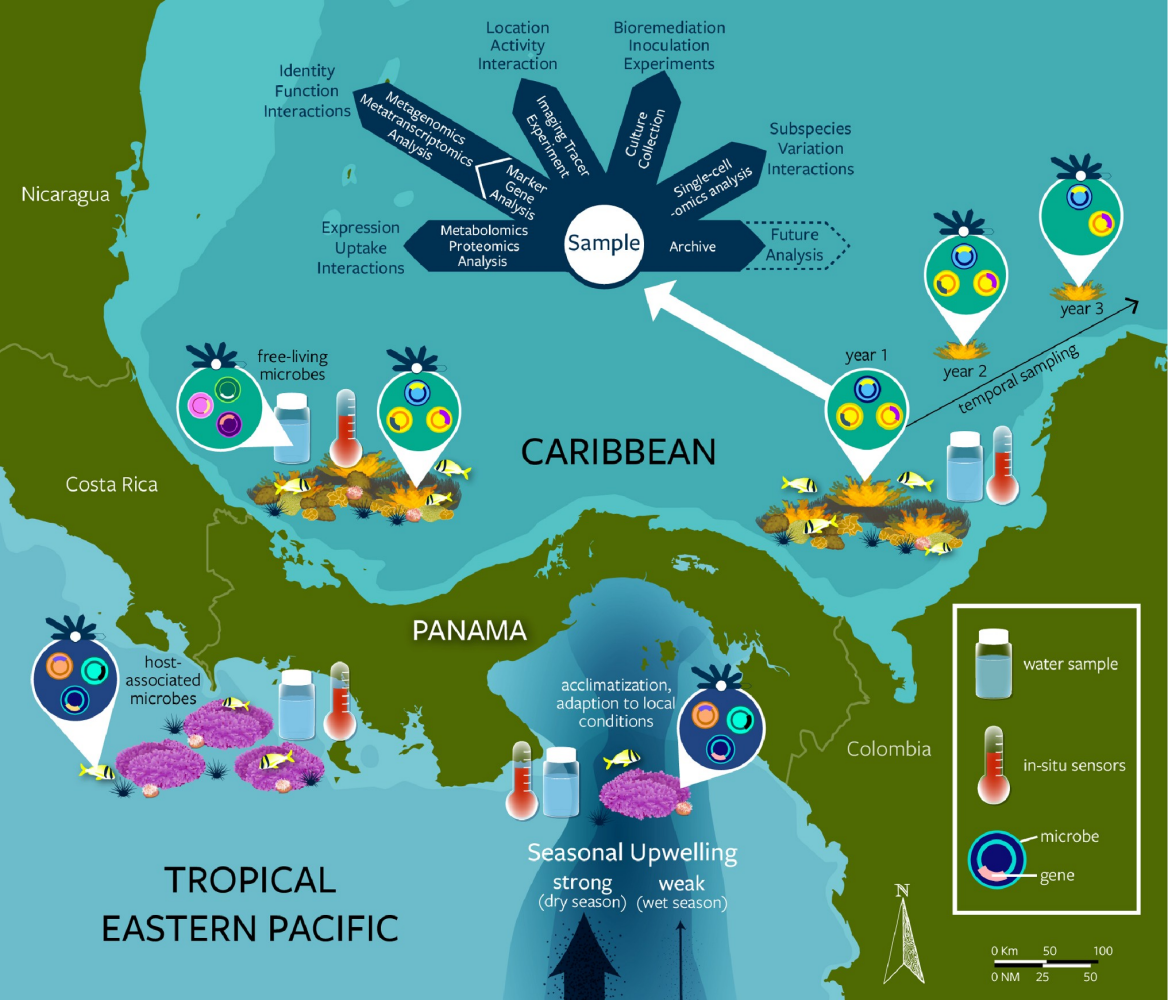
Methodological approach to leveraging a natural experiment, the Isthmus of Panama, for the long-term study of host–microbe ecology and evolution. Present-day organisms physically separated by the Isthmus of Panama are adapted to the distinct environmental conditions of the productive TEP and the oligotrophic Caribbean. In the Gulf of Panama of the TEP, organisms experience some of the most drastic annual fluctuations in temperature, pH, oxygen, salinity, and nutrients, due to intense seasonal upwelling. Conversely, the nearby Gulf of Chiriquí of the TEP experiences weak to no upwelling due to trade winds being largely blocked by the Cordillera Central mountain range. Multidisciplinary and long-term research on hosts and associated microbes across these environmental spatiotemporal gradients, where decades of taxonomic, ecological, and evolutionary research can be leveraged, will help capture the dynamics of host–microbe interactions. TEP, Tropical Eastern Pacific.

The formation of the Isthmus of Panama presents an unparalleled opportunity for exploring the roles of biogeographic isolation and environmental change in structuring host-associated microbes (Fig 4). In the Miocene, populations of marine organisms and their microbial symbionts moved freely between the Tropical Eastern Pacific (TEP) and Caribbean in a large, unified tropical faunal province dominated by high primary productivity and seasonal upwelling [64]. Gradually, over millions of years, this shared faunal province became severed by uplift of the Isthmus of Panama, which finally closed approximately 2.8 Ma (million years ago) [65]. The Caribbean became nutrient poor, causing widespread extinction and a concurrent proliferation of coral reefs and immigration of new biotas [66]. In contrast, the TEP continued to experience strong seasonal upwelling and nutrient-rich conditions. In many cases, closely related animal hosts diverged and followed separate evolutionary trajectories, adapting to the strongly contrasting environments on opposite sides of the Isthmus. Presumably, their associated microbiomes did so too. Today’s Caribbean and TEP marine ecosystems of Panama and Central America are home to hundreds of sister species that emerged through transisthmian vicariance, representing all major taxonomic groups. Decades of research have identified phylogenetic relationships between hosts, as well as the behavioral, physiological, and genetic mechanisms involved in host divergence and reproductive isolation [65]. These data place host-associated microbes into an unrivaled ecological and evolutionary framework.

Ocean gateways that remain open today also present unique attributes suitable for natural experiments. The narrow Strait of Bab al Mandab connects the warm and saline semi-enclosed Red Sea with the open and more variable Arabian Sea. The Red Sea is host to many endemic species (5% to 13% endemic across a range of taxa [67]), while the pronounced seasonal variations in the Arabian Sea have driven fine-scale local adaptations [68]. Although the Mediterranean has been connected to the Atlantic through the Strait of Gibraltar since the end of the Messinian Salinity Crisis 5.3 Ma [69], the modern Mediterranean fauna bears the more recent imprint of Pleistocene glacial and interglacial cycles. Temperature shifts in the basin over the last 2 to 3 Million years dictated whether subtropical or higher latitude taxa could successfully colonize the basin from the Atlantic and subsequent basin wide extinctions [70]. The historical context of these ocean gateways and their impacts on gene flow have been explored in a myriad of organisms ranging from plants to invertebrates, fish, and mammals.

Other important biogeographic regions characterized by unique environmental conditions, long-term data collection, and good scientific infrastructure include the Great Barrier Reef [71], the Baltic Sea [72], the Larsen B ice shelf [73], Ischia Island [74], and the French Polynesian island of Moorea [75] (Fig 1). Extensive research networks such as the National Science Foundation’s Long-Term Ecological Research (LTER) Network, the Smithsonian Institution’s Marine Global Earth Observatory (MarineGEO) network of partners, and the Marine Biodiversity Observation Network (MBON; Fig 1) are set to play a fundamental role in providing researchers with logistical access (field labs and sites) to these marine ecosystems and rigorously collected physicochemical and biological contextual data [via, for example, long-term deployment of sondes (CTDs) and data loggers, standardized visual surveys, and other methods] at a global scale (Fig 1). The many examples of crucial long-term support networks typically overlook host-associated microbes. They can serve as a good model going forward or they could be leveraged to facilitate comparative studies that map microbial variation across communities of hosts from unique marine ecosystems to help us elucidate how host–microbe associations adjust to changes in their environment at multiple temporal (from seasonal to geological) and spatial scales (from local to biogeographical; Fig 4).

### Emergence of volcanic islands

Novel habitats such as remote island archipelagos that formed over relatively recent geological history also offer exceptional opportunities to study evolutionary processes in marine and terrestrial host-associated mutualistic microbes. Initially barren, shallow coastal areas were colonized by marine organisms from neighboring areas that subsequently evolved in conditions that are often drastically different from their native environments. Three archipelagos in particular, Hawai’i and Marquesas, located at the periphery of the Indo-Pacific region, and the Galapagos in the TEP, have provided tremendous opportunities to study evolution through comparative phylogeography (Fig 1). All three are composed of young islands (25 to 0.75 Ma, 5.5 to 0.4 Ma, and 3.2 to 0.05 Ma, respectively; reviewed in [76]) with high proportions of endemic species (25.0% [77], 13.7% [78], and 13.6% [79] for fishes, respectively). The shallow coastal habitats of the islands within these archipelagos were colonized sequentially by marine species as they formed, resulting in a “progression” pattern whereby evolutionarily older lineages consistently occur on older islands [80]. These regions provide a unique historical context for understanding the evolution of host-associated microbes and their roles in driving host ecological success when new ecological opportunities emerge.

### Ongoing human-induced changes

Marine communities are changing rapidly in the face of climate change and other anthropogenic activities [81]. The physicochemical parameters associated with the catastrophic changes occurring over contemporary timescales are now relatively well characterized, but the effects on most host-associated microbes are still virtually unknown [82]. Coral bleaching is a notable exception. As host species and their associated microbes shift in distribution, they often face novel abiotic and biotic conditions. For example, melting of ice is opening new pathways for the movement of animals, plants, and microbes through the Arctic, from the North Pacific to the North Atlantic, leading to one of the largest species invasions ever observed [83]. The gradual increase in salinity caused by the expansion of the Panama Canal, along with predicted increased runoff and evaporation, will likely result in greater movement of marine species between the tropical Western Atlantic and the TEP [84] (Fig 4). Construction of the Suez Canal in 1869 caused an influx of saline water into the Mediterranean that was followed by the intrusion of invasive species from the subtropical Red Sea [85]. Rats introduced to islands of the Chagos Archipelago precipitated a decline in bird density, thereby reducing the nitrogen input on land and in the sea with downstream effects on coral reef productivity [86]. Finally, many tropical species are expanding their distributions with the warming climate [87]. For example, mangrove trees take advantage of the lower frequency of freezes to colonize salt marshes [88], which allows many invertebrate and fish species to simultaneously expand their ranges. Additional anthropogenic pressures stem from episodic or localized disasters such as the 2010 Deepwater Horizon oil spill in the Gulf of Mexico [89], anoxic events (Bocas del Toro [90]), sediment runoff events (Great Barrier Reef [91]), as well as water pollution and eutrophication around large urban centers such as Jakarta, Hong Kong, and Singapore [92] (Fig 1). These anthropogenic changes provide multiple opportunities to understand how the rapid evolutionary potential of host-associated microbes underpins adaptive evolution in hosts.

## Conclusions

Understanding what changes in host-associated microbes mean for the maintenance of marine communities and ecosystems requires measurements that go far beyond the typical life span of a publicly funded scientific project. The integration of microbial sampling into long-term ecological monitoring programs across key geographic locations will help us identify important core and transient host-associated microbes and provide the fundamental basis for mechanistic studies. Researchers should focus on the vast majority of marine animals and plants that are able to interchange microbial partners, genes, and functions with surrounding microbial communities. The future of marine ecosystems around the globe may in part depend upon the ability of marine organisms to dip into the enormous pool of microbes and harness their remarkable genetic potential.

## Abbreviations

LTER: Long-Term Ecological Research
MarineGEO: Marine Global Earth Observatory
MBON: Marine Biodiversity Observation Network
TEP: Tropical Eastern Pacific
